# Effect of proton pump inhibitors on sympathetic hyperinnervation in infarcted rats: Role of magnesium

**DOI:** 10.1371/journal.pone.0202979

**Published:** 2018-08-28

**Authors:** Tsung-Ming Lee, Nen-Chung Chang, Shinn-Zong Lin

**Affiliations:** 1 Cardiovascular Institute, An Nan Hospital, China Medical University, Tainan, Taiwan; 2 Department of Medicine, China Medical University, Taichung, Taiwan; 3 Department of Internal Medicine, School of Medicine, Taipei Medical University, Taipei, Taiwan; 4 Division of Cardiology, Department of Internal Medicine, Taipei Medical University Hospital, Taipei, Taiwan; 5 Department of Neurosurgery, Buddhist Tzu Chi General hospital, Tzu Chi University, Hualien, Taiwan; Scuola Superiore Sant'Anna, ITALY

## Abstract

The long-term use of proton pump inhibitors (PPIs) has been shown to increase the risk of cardiovascular mortality, however the molecular mechanisms are unknown. Superoxide has been implicated in the regulation of nerve growth factor (NGF), a mediator of sympathetic innervation. The purpose of this study was to determine whether PPIs increase ventricular arrhythmias through magnesium-mediated superoxide production in infarcted rats. Male Wistar rats were randomly assigned to receive vehicle, omeprazole, omeprazole + magnesium sulfate, or famotidine treatment for 4 weeks starting 24 hours after the induction of myocardial infarction by ligating the coronary artery. Increased myocardial superoxide and nitrotyrosine levels were noted post-infarction, in addition to a significant upregulation of NGF expression on mRNA and protein levels. Sympathetic hyperinnervation after infarction was confirmed by measuring myocardial norepinephrine and immunofluorescent analysis. Compared with the vehicle, omeprazole-treated infarcted rats had significantly reduced myocardial magnesium content, increased oxidant production, and increased sympathetic innervation, which in turn increased ventricular arrhythmias. These effects were prevented by the coadministration of magnesium sulfate. In an *in vivo* study, an omeprazole-induced increase in NGF was associated with a superoxide pathway, which was further confirmed by an *ex vivo* study showing the attenuation of NGF levels after coadministration of the superoxide scavenger Tiron. Magnesium sulfate did not further attenuate NGF levels compared with omeprazole + Tiron. Our results indicate that the long-term administration of PPIs was associated with reduced tissue magnesium content and increased myocardial superoxide production, which exacerbated ventricular arrhythmias after infarction. Magnesium may be a potential target for PPI-related arrhythmias after infarction.

## Introduction

Proton pump inhibitors (PPIs) are frequently used to prevent or treat peptic ulcers, especially in patients with acute coronary syndrome who need dual antiplatelet treatment. However, their safety has not been approved by regulatory authorities after myocardial infarction (MI). A few epidemiological studies have reported inconsistent results regarding the association between the use of PPIs and cardiovascular events. A recent data-mining study suggested that PPIs may be associated with an elevated risk of MI in the general population [1]. In contrast, clinical studies have not shown an association between the use of PPIs and cardiovascular events [2], and it appears that confounding factors such as the patients’ medications, lifestyle, comorbidities and genetic background should be taken into account when evaluating these data.

Data on PPIs and hypomagnesemia are inconsistent and conflicting. Many observational studies have shown a positive association between the long-term use of PPIs and hypomagnesemia [3,4]. However, others have not reported any differences in serum magnesium levels between PPI users and non-PPI users [5]. Hypomagnesemia can lead to a decrease in glutathione concentration and lower activities of superoxide dismutase in red blood cells [6]. Given that a negative correlation has been reported between magnesium levels and plasma superoxide anions [7], it is important to determine whether the use of PPIs is associated with hypomagnesemia.

We previously showed that pharmacological interventions to scavenge reactive oxygen species (ROS) can ameliorate sympathetic innervation after MI [8–11]. Regional sympathetic hyperinnervation has often been observed at the border zone during the chronic stage of MI [11], and has been associated with lethal arrhythmias [12]. Nerve growth factor (NGF), a prototypical growth factor of the neurotrophin family, plays an essential role in the differentiation, survival, and synaptic activity of the peripheral sympathetic nervous systems [13]. The *NGF* promoter contains a functional activator protein-1 site [14], which is subjected to the redox states of its regulatory cysteine residue [15]. Peroxynitrite, the reaction product of nitric oxide (•NO) and superoxide (O_2_^•−^), is a potent oxidant and nitrating agent. Previous studies have shown that peroxynitrite is an important trigger of NGF formation and a brief exposure to peroxynitrite can increase *NGF* expression [16]. Whether PPIs can affect sympathetic innervation via hypomagnesemia-mediated increases in oxidative stress is unclear. Therefore, this study aimed 1) to assess whether the chronic administration of a PPI, omeprazole with a therapeutically relevant dose, could exaggerate heart reinnervation through an increase in the expression of NGF, 2) to evaluate the antioxidation effect of Mg^2+^, and 3) to investigate the functional significance of changes in the sympathetic reinnervation by programmed electrical stimulation in a rat model of MI.

## Methods

### Animals

Male Wistar rats were purchased from LASCO (Taipei, Taiwan). All experiments were conducted according to protocols approved by the China Medical University Committee on Animal Care of (protocol number #2016–071). Two to three rats were housed in temperature-controlled ventilated cabinets and monitored daily for any signs of distress or clinical symptoms of illness by trained personnel. At the end of the experiment, the rats were sacrificed under sodium pentobarbitone anesthesia according to the guidelines for euthanasia.

**Fig 1:**
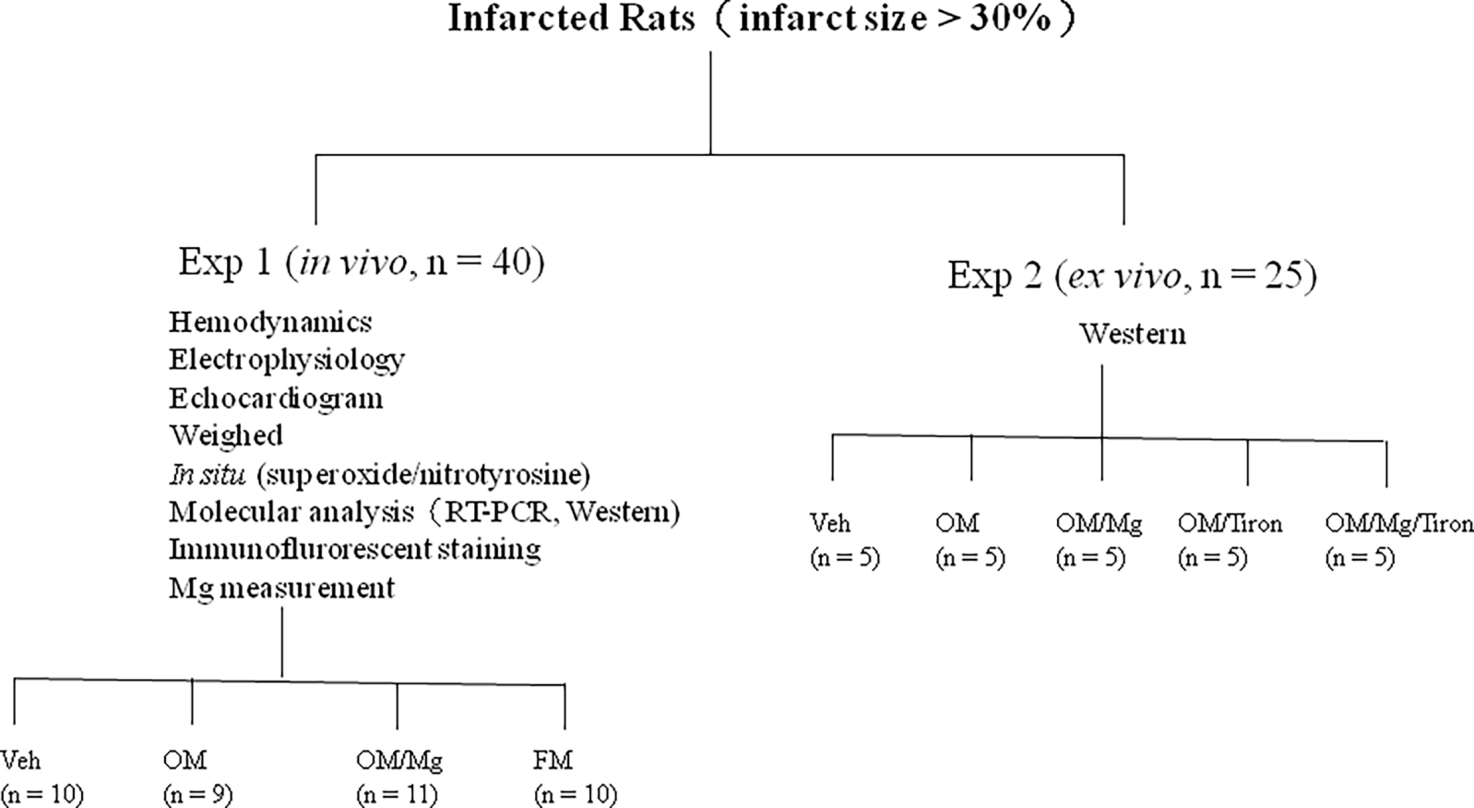
Flow chart for rats with infarct size >30%.

Experiment 1 (*in vivo*). Eight-week-old rats (220–250 g) were subjected to ligation of the anterior descending artery after being anesthetized with ketamine-xylazine (90 mg/kg-9 mg/kg, intraperitoneally) as previously described [10] resulting in myocardial free wall infarction of the left ventricle (LV). The rats were then randomized into four groups as follows (Fig 1): vehicle (saline), omeprazole (5 mg/kg body weight per day), omeprazole + magnesium sulfate (107 mg/kg body weight per day), or famotidine (an H2 receptor blocker, 3 mg/kg body weight per day). The dose of omeprazole was based on an oral human dose of 40 mg normalized to body surface area, and it was smaller than previously reported study [17]. The doses of magnesium sulfate [18] and famotidine [19] have been shown effective modulation of the biological activities. The rats were kept in cabinets controlled for temperature, humidity, and light. They were fed with standard chow containing magnesium (0.23% w/w), and water was given ad libitum.

The drugs were started 24 hours post-infarction to maximize their effects [20]. The duration of the study was set at 4 weeks, as the myocardial remodeling process in rats has been reported to be mostly complete (70–80%) within 3 weeks [20]. The rats were given the drugs daily by oral gavage. The drugs were stopped in each group approximately 24 hours before the end of the experiments to stop their pharmacological effects.

Experiment 2 (*ex vivo*). To further confirm the mechanism by which PPIs act as oxidants, a superoxide scavenger (Tiron, 4,5-dihydroxy-1,3-benzenedisulfonic acid, Sigma) was used in an *ex vivo* model. Four weeks after coronary ligation-induced MI, the infarcted rat hearts were isolated, divided into four groups, and subjected to the following treatment: vehicle, omeprazole (2 mg/ml), omeprazole + magnesium sulfate (2 mM), omeprazole + Tiron (100 μM), and omeprazole + magnesium sulfate + Tiron. These doses of magnesium sulfate [21] and Tiron [22] have been shown to be effective in modulating biological activities. In a clinical setting, the physiological concentration of serum magnesium is around 1 mM, however it can increase to 3 mM after magnesium treatment [21]. The hearts were perfused with MgCl_2_ 1.0 mM as described previously [23]. At the end of the study, myocardial peroxynitrite was measured in all hearts (n = 5 in each group), and Western blot analysis was used to measure NGF protein at the border zone (< 2 mm within the infarct).

### Echocardiography

Twenty-eight days after the operation, the rats were lightly anesthetized with intraperitoneal injections of ketamine HCl (25 mg/kg). Echocardiographic measurements were performed using a GE Healthcare Vivid 7 Ultra-sound System (Milwaukee, WI) equipped with a 14-MHz probe as previously described [23]. LV end-diastolic diameter dimension (LVEDD) and LV end-systolic diameter dimension (LVESD) were measured from M-mode tracings of the LV via a parasternal long-axis view, and fractional shortening (FS) (%) was calculated according to the American Society for Echocardiology. All measurements were averaged over five consecutive cardiac cycles. All analyses were conducted by an experienced observer blinded to treatment group allocation. Hemodynamic measurements of the hearts were then rapidly performed after systemic heparinization.

### Hemodynamic and infarct size measurements

After the anesthetized rats had been given an additional intraperitoneal dose of ketamine-xylazine (90 mg/kg-9 mg/kg), hemodynamic parameters were measured at the end of the echocardiogram. A polyethylene Millar catheter connected to a transducer (Model SPR-407, Miller Instruments, Houston, TX) was inserted into the LV of each rat, and measurements of the LV systolic and diastolic pressure were averaged from five consecutive pressure cycles as previously described [23]. The maximum rates of LV pressure increase (+dP/d*t*) and decrease (-dP/d*t*) were then measured. After arterial pressure measurements had been taken, the atria and right ventricle were trimmed off. The LV was then washed in ice-cold isotonic saline solution, weighed, and immediately frozen in liquid nitrogen after a coronal section had been obtained. This section was fixed in 10% formalin, embedded in paraffin, stained with hematoxylin and eosin and trichrome, and the infarct size was measured as previously described [23]. In consideration of the clinical significance, only rats with a large infarction (>30%) were chosen in this analysis.

### *Ex vivo* electrophysiological studies

The functional significance of sympathetic innervation was assessed by programmed electrical stimulation to identify the potential arrhythmogenic risk. After perfusing the isolated hearts with a modified Tyrode’s solution, they were observed for 20 minutes to allow for stabilization of rhythm and contraction. As residual neural plasticity at the infarct site has been shown to be a determinant of the response to program-induced ventricular arrhythmias [24], only the rats in which the infarcted area of the LV had been fully replaced by scar tissue were included. Electrodes were sewn onto the epicardial surfaces of right ventricular outflow tracts of the rats, and programmed electrical stimulation was performed. Ventricular arrhythmias were induced using a Bloom stimulator (Fischer Medical Technologies, Broomfield CO) with eight paced beats and a 120-ms cycle length (S1) followed by one to three additional stimuli (S2, S3, and S4) with shorter coupling intervals. The ventricular pacing endpoint was defined as when ventricular tachyarrhythmias had been induced. A ventricular tachyarrhythmia (including ventricular tachycardia and ventricular fibrillation) lasting 15 beats or less was considered to be non-sustained, whereas a ventricular tachyarrhythmia lasting more than 15 beats was defined as being sustained. We used a modified arrhythmia scoring system as previously described [25]. In hearts with more than one type of arrhythmia, only the highest score was used. All of the experiments were usually completed within 10 minutes, and pilot studies showed no significant tissue edema during this period.

### Immunofluorescent studies of tyrosine hydroxylase and growth associated protein 43 (GAP43)

Immunohistochemical staining for tyrosine hydroxylase (a selective axon marker) and GAP43 (a marker peptide for neuronal regeneration and outgrowth [26]) was performed on LV muscle from the border regions to investigate the quantification of sympathetic nerve fibers. As variable sympathetic innervation has been reported in papillary muscles, they were excluded from this study [27]. Five-μm-thick paraffin-embedded sections were prepared, and the sections were incubated with anti-tyrosine hydroxylase (1:200; Chemicon, CA, USA) and anti-GAP43 (1:400; Chemicon, CA, USA) antibodies in 0.5% BSA in PBS overnight at 37°C. Monoclonal goat anti-mouse IgG conjugated to fluorescein isothiocyanate was used as the second antibody for tyrosine hydroxylase, and conjugated to rhodamine for GAP43. Directly conjugated antibodies with identical isotypes were used as negative controls.

Nerve fiber density was estimated qualitatively from 10 randomly selected fields at 400× magnification via computerized planimetry tracings (Image Pro Plus, CA) as described previously [28], with values expressed as the ratio of labeled nerve fiber area to total area. The investigator was blinded to the various experiments.

### *In situ* detection of superoxide and nitrotyrosine

Dihydroethidium (DHE, 1 μM, Invitrogen Molecular Probes, Eugene, OR, USA) fluorescence was used to evaluate *in situ* formation of myocardial intracellular superoxide production. Five-μm-thick sections of paraffin-embedded tissues were incubated with DHE in PBS (10 mM) at 37°C in a dark and humidified container for 30 minutes. The presence of superoxide radicals in the tissue was assessed using red fluorescence, and the density was recorded as arbitrary units per square mm field.

Nitrative stress was assessed by detecting nitrotyrosine in LV myocardial sections with immunohistochemistry at the border zone. Nitrotyrosine has been reported to be a biomarker for the formation of peroxynitrite, a by-product of ^•^NO and O_2_^•−^. After antigen retrieval and quenching endogenous peroxidase, the slides were immunostained for nitrotyrosine using a rabbit polyclonal nitrotyrosine antibody (1:200, Millipore, Bedford, MA, USA) overnight at 4°C.

### Western blot analysis of NGF

Western blotting was performed to quantify the levels of NGF from the myocardium at the border zone as previously described [23]. The primary antibodies were NGF (Chemicon, CA, USA) and β-actin (Santa Cruz Biotechnology). All of the experiments were performed in triplicate, and results were expressed as the mean value.

### Real-time RT-PCR of NGF

Samples obtained from the border zone were subjected to real-time quantitative RT-PCR using a TaqMan system (Prism 7700 Sequence Detection System, PE Biosystems). The primers for *NGF* were 5′-CACACTGAGGTGCATAGCGT-3′ (sense) and 5′-TGATGACCGCTTGCTCCTGT-3′ (antisense), and for *cyclophilin* the primers were 5′-ATGGTCAACCCCACCGTGTTCTTCG-3′ (sense) and 5′-CGTGTGAAGTCACCACCCTGACACA-3′ (antisense). We used *cyclophilin* mRNA as the internal standard because it has been shown to be expressed at fairly constant levels in most tissues. The expression was quantified by normalization to the expression of the housekeeping gene *cyclophilin*. The reaction conditions were set on a computer that was connected to the detector for the 40 cycles of the amplification step.

### Laboratory measurements

Blood samples from the aorta were immediately centrifuged at 3,000 *g* for 10 min, and the serum was stored at -70°C until further analysis. Serum Mg^2+^ concentrations were determined using an automated chemical analyzer.

Myocardium at the border zone was processed according to the manufacturer’s instructions. The Mg^2+^ content of the myocardium was assessed by chopping the LV and digesting them with nitric acid (Sigma), followed by measurements using an atomic absorption spectrophotometer (Shimadzu, Tokyo, Japan) as described previously [29]. The total level of norepinephrine in the myocardium was measured using a commercial ELISA kit (Noradrenalin ELISA, IBL Immuno-Biological Laboratories, Hamburg, Germany). Myocardial peroxynitrite formation was estimated by measuring the level of free nitrotyrosine (as a marker for peroxynitrite formation) using an ELISA kit (Cayman Chemical, Ann Arbor, MI, USA) in myocardial homogenates.

Superoxide production at the border zone was also measured using lucigenin (5 μmol/L bis-N-methylacridinium nitrate, Sigma, St. Louis, MO) and enhanced chemiluminescence as previously described [28]. The specific chemiluminescence signal was calculated after subtraction of the background signal and expressed as counts per minute per milligram weight (cpm/mg).

### Statistical analysis

The results are presented as mean ± SE. Statistical analysis was conducted with the SPSS statistical package (SPSS, version 18.0, Chicago, Illinois). Group differences were determined by a one-way ANOVA. A multiple comparison test (Scheffe’s method) was performed if significant differences between the two groups were observed. A non-parametric Kruskal Wallis test was used to analyze the differences of electrophysiological data (scoring of programmed electrical stimulation-induced arrhythmias) followed by a Mann-Whitney test. A *P* value of < 0.05 was considered to indicate statistical significance.

## Results

### PPI treatment impaired ventricular remodeling

Four weeks after infarction, the anterior wall of the infarcted LV was replaced completely by a thin layer of collagenous scar tissue. The weight of the LV inclusive of the septum remained essentially unaltered for 4 weeks among the infarcted groups (Table 1). The ratios of right ventricular weight/ tibia length and lung weight/ tibia length, indexes of lung edema, were significantly higher in the omeprazole-treated infarcted group than in the vehicle-treated infarcted group. The ratios were attenuated after the addition of magnesium sulfate. Unlike omeprazole, famotidine did not affect remodeling of the right ventricle or lung after infarction. The values of +dP/d*t* and -dP/d*t* were significantly decreased in the infarcted group treated with omeprazole, which could be improved after the coadministration of magnesium sulfate. There were no significant differences in LV end-systolic pressure, LV end-diastolic pressure, and infarct size among the infarcted groups.

**Table 1:** Cardiac morphology, hemodynamics, and Mg ^2+^ and NE concentrations at the end of study.

	Sham	Infarction			
Parameters	Vehicle	Vehicle	OM	OM + Mg	FM
No. of rats	10	10	9	11	10
Body weight, g	322 ± 11	305 ± 13	315 ± 11	320 ± 10	316 ± 12
Heart rate, bpm	399 ± 12	418 ± 12	407 ± 15	398± 14	402 ± 13
LVESP, mm Hg	104 ± 7	99 ± 10	94 ± 12	98 ± 10	102 ± 7
LVEDP, mm Hg	7 ± 4	18 ± 3*	19 ± 4*	16 ± 5*	17 ± 4*
+dP/d*t*, mm Hg/s	7252 ± 328	2362 ± 304*	2148 ± 254*†	2528 ± 302*‡	2601 ± 264*‡
-dP/d*t*, mm Hg/s	6982 ± 306	2102 ± 274*	1754 ± 248*†	2363 ± 273*‡	2352 ± 314*‡
Infarct size, %	—	40 ± 3	41 ± 3	40 ± 3	41 ± 3
LVW/BW, mg/g	2.05 ± 0.24	3.12 ± 0.27*	2.99 ± 0.22*	2.85 ± 0.31*	3.08 ± 0.32*
RVW/BW, mg/g	0.45 ± 0.16	0.68 ± 0.13*	0.85 ± 0.21*†	0.68 ± 0.18*‡	0.65 ± 0.16*‡
LungW/BW, mg/g	3.75 ± 0.32	5.57 ± 0.28*	6.86 ± 0.35*†	5.76 ± 0.28*‡	5.21 ± 0.38*‡
Serum Mg^2+^, mg/dl	1.81 ± 0.08	1.89 ± 0.12	1.83 ± 0.11	1.85 ± 0.14	1.92 ± 0.13
LV Mg ^2+^, μmol/g dry weight	36.1 ± 2.6	35.1 ± 2.5	29.4 ± 2.8*†	38.9 ± 3.2*‡	36.4 ± 2.9*‡
LV NE, μg/g protein	1.21 ± 0.06	2.49 ± 0.15*	3.14 ± 0.18*†	1.79 ± 0.12*‡	1.84 ± 0.15*‡

Values are mean ± SE. BW, body weight; FM, famotidine; LungW, lung weight; LVEDP, left ventricular end-diastolic pressure; LVESP, left ventricular end-systolic pressure; LVW, left ventricular weight; NE, norepinephrine; OM, omeprazole; RVW, right ventricular weight.

*P < 0.05 compared with sham-operated rats

†*P* < 0.05 compared with the infarcted group treated with vehicle

**‡***P* < 0.05 compared with the infarcted group treated with OM.

In a comparison with sham-operated animals, the MI hearts showed echocardiographically-derived structural and functional changes. The structural changes after infarction included increased LV diastolic and systolic diameters (Table 2), consistent with LV remodeling. Echocardiography showed significant increases in LVESD and LVEDD in the omeprazole-treated infarcted rats compared with the vehicle-treated infarcted rats. LV fractional shortening was significantly decreased in the omeprazole-treated infarcted rats compared with the vehicle-treated infarcted rats (16 ± 2% vs. 20 ± 2% in the vehicle group, *P* < 0.05). Omeprazole significantly increased hypertrophy of the remote myocardium (i.e. LV posterior wall, *P* < 0.05) but did not affect the thickness of the infarcted part (i.e. interventricular septum). The infarcted group treated with omeprazole and magnesium sulfate had improved LV remodeling and function. There were no significant differences in echocardiographic parameters between the famotidine-treated infarcted rats and vehicle-treated infarcted rats.

**Table 2:** Echocardiographic findings at the end of study.

	Sham	Infarction			
Parameters	Vehicle	Vehicle	OM	OM + Mg	FM
No. of rats	10	10	9	11	10
IVS (mm)	1.4 ± 0.1	0.4 ± 0.2*	0.5 ± 0.2*	0.5 ± 0.1*	0.5 ± 0.1*
LVEDD (mm)	5.6 ± 0.2	8.5 ± 0.2*	9.4 ± 0.2*†	8.3 ± 0.1*‡	8.7 ± 0.2*‡
LVESD (mm)	3.5 ± 0.2	6.8 ± 0.2*	7.9 ± 0.2*†	6.5 ± 0.3*‡	6.9 ± 0.2*‡
LV posterior wall (mm)	1.4 ± 0.1	1.7 ± 0.2*	2.0 ± 0.2*†	1.8 ± 0.2*‡	1.8 ± 0.2*
FS (%)	38 ± 2	20 ± 2*	16 ± 2*†	22± 3*‡	19 ± 2*

Values are mean ± SE. Abbreviations as in Table 1. FS, fractional shortening; IVS, interventricular septum; LVEDD, left ventricular end-diastolic dimension; LVESD, left ventricular end-systolic dimension

*P < 0.05 compared with the sham group

†*P* < 0.05 compared with the infarcted group treated with vehicle

**‡***P* < 0.05 compared with the infarcted group treated with OM.

### PPI treatment increased myocardial superoxide and nitrotyrosine levels

**Fig 2:**
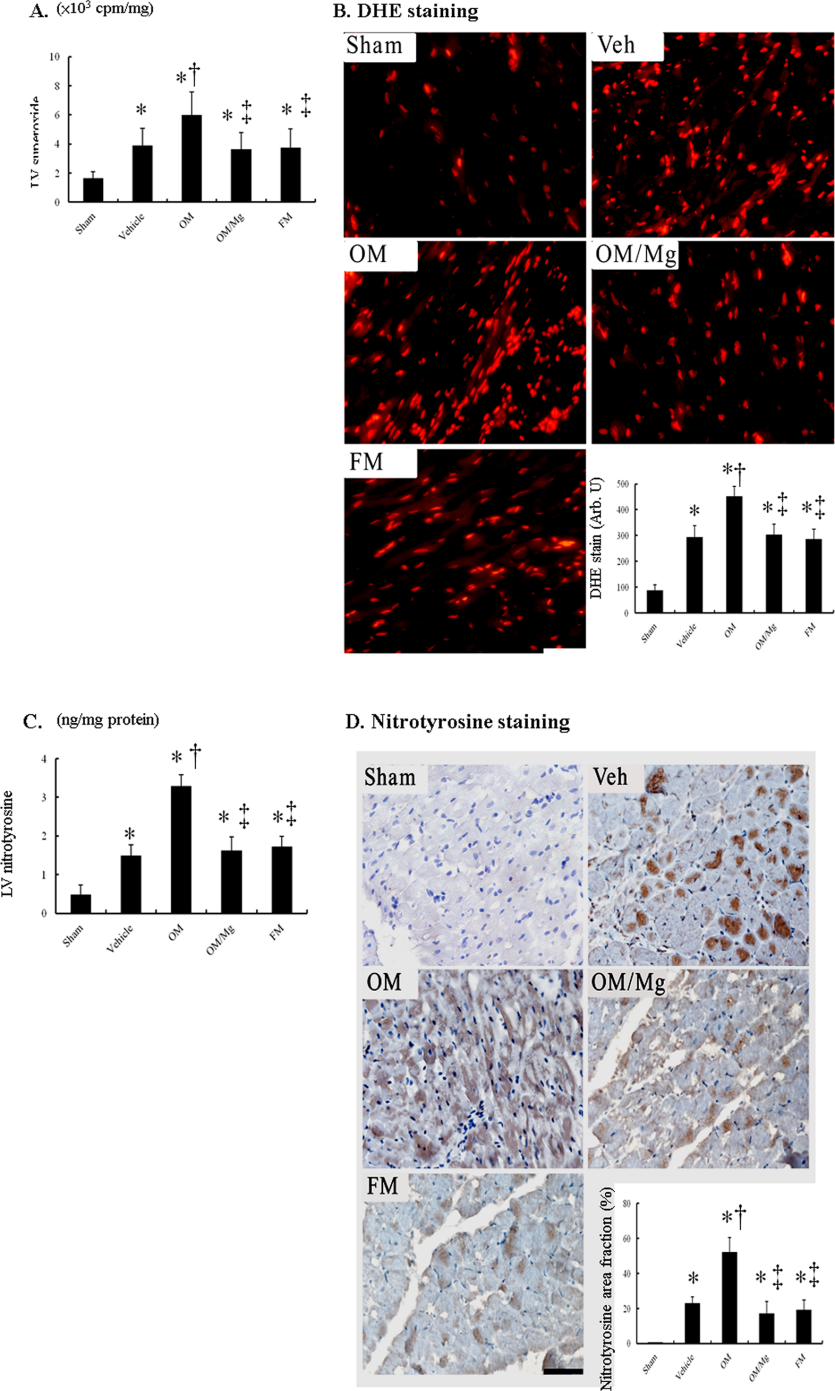
In vivo myocardial superoxide and nitrotyrosine levels. Myocardial (A) superoxide was assessed by chemiluminescence, (B) DHE staining and quantitative analysis, (C) nitrotyrosine by ELISA, and (D) nitrotyrosine immunoreactive staining and quantitative analysis from the border zone. Myocardial DHE (red fluorescence) and nitrotyrosine (brown) staining showed more intense signals (nuclear position for DHE and cytoplasm for nitrotyrosine) after MI. Compared with the vehicle, myocardial superoxide and nitrotyrosine levels in the OM-treated infarcted group were significantly increased, and this could be significantly reduced after adding MgSO_4_. Bar = 50 μm. Sham (n = 10); Vehicle (n = 10); OM, omeprazole (n = 9); OM/MgSO_4_ (n = 11); FM, famotidine (n = 10). *P < 0.05, compared with the sham group; †*P* < 0.05, compared with the vehicle-treated rats; ‡*P* < 0.05, compared with the OM-treated rats.

Superoxide production as assessed by chemiluminescence was significantly increased in the border LV tissues from the vehicle-treated infarcted rats compared with the sham-operated rats, and was further increased in the omeprazole-treated infarcted rats (Fig 2A). The addition of magnesium sulfate significantly decreased superoxide production. Famotidine did not affect myocardial superoxide production compared with the vehicle.

Hydroethidine (HE) or dihydroethidium (DHE), a redox-sensitive probe, has been widely used to detect intracellular superoxide anion Hydroethidine (HE) or dihydroethidium (DHE), a redox-sensitive probe, has been widely used to detect intracellular superoxide anion dihydroethidium (DHE), a redox-sensitive probe, has been widely used to detect intracellular superoxide anion dihydroethidium (DHE), a redox-sensitive probe, has been widely used to detect intracellular superoxide anionAs shown in Fig 2B, post-infarction remodeling markedly enhanced the intensity of DHE staining at the border zone in the vehicle-treated rats compared with the sham-operated rats. In addition, compared with the vehicle-treated infarcted rats, the omeprazole-treated infarcted rats had significantly increased fluorescence. This increased intensity of the fluorescent signals after infarction could be significantly attenuated by administering magnesium sulfate.

The level of myocardial nitrotyrosine was significantly higher in the vehicle-treated infarcted rats than in the sham-operated rats (*P <* 0.01, Fig 2C). Similarly, nitrotyrosine immunoreactivity, as assessed by increased brown staining (Fig 2D), was significantly increased after MI, and further increased after treatment with omeprazole. The increased fluorescence in the omeprazole-treated infarcted group could be reversed by adding magnesium sulfate.

#### PPI treatment increased the expressions of NGF protein and mRNA

**Fig 3:**
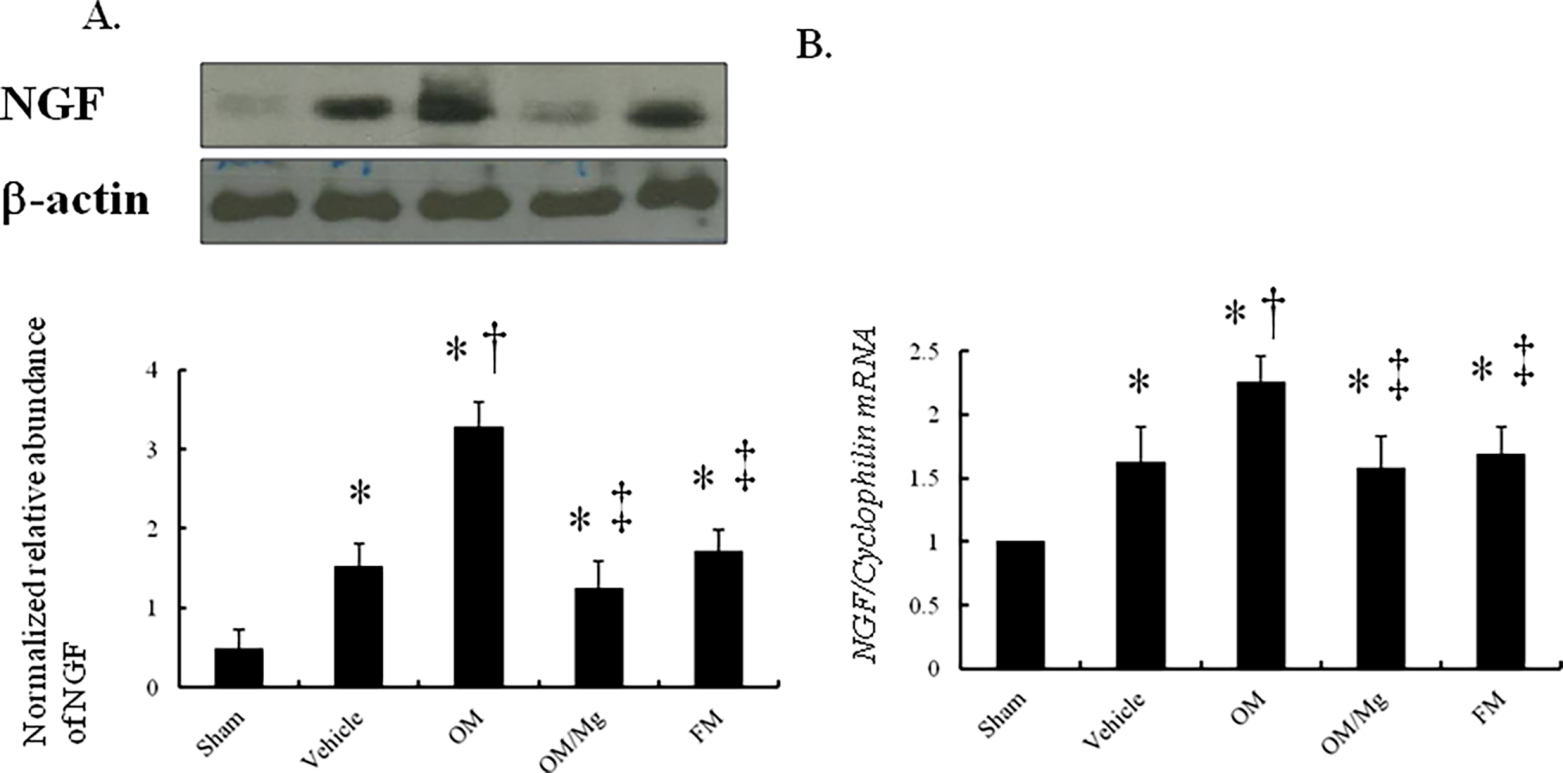
Western blot and RT-PCR of NGF in the *in vivo* study. (A) Compared with the vehicle-treated infarcted rats, the omeprazole-treated infarcted rats had significantly higher NGF levels by quantitative analysis. Relative abundance was obtained by normalizing the density of NGF proteins to that of β-actin. Results are mean ± SE of three independent experiments. (B) Each mRNA was corrected for an mRNA level of *cyclophilin*. sham (n = 10); vehicle (n = 10); omeprazole (n = 9); omeprazole/MgSO_4_ (n = 11); famotidine (n = 10). *P < 0.05, compared with the sham group; †*P* < 0.05, compared with the vehicle-treated rats; ‡*P* < 0.05, compared with the OM-treated rats.

Western blot analysis showed that NGF levels were significantly upregulated by 3.08-fold at the border zone in the vehicle-treated infarcted rats compared to the sham-operated rats (*P* < 0.0001, Fig 3A). Compared with the vehicle-treated infarcted rats, the NGF levels were significantly increased at the border zone in the omeprazole-treated rats. After the coadministration of magnesium sulfate, the NGF levels were significantly lower compared with omeprazole alone.

PCR amplification of cDNA revealed that the *NGF* mRNA expression was upregulated by 1.62-fold at the border zone in the vehicle-treated rats compared with the sham-operated rats (*P* < 0.0001, Fig 3B). In addition, the *NGF* mRNA expression was significantly increased in the omeprazole-treated infarcted rats compared to the vehicle-treated rats, and this could be reversed after adding magnesium sulfate.

### PPI treatment increased myocardial norepinephrine levels and sympathetic innervation

To assess cardiac sympathetic function, we determined the levels of LV norepinephrine, and found that they were significantly upregulated by 2.08-fold at the border zone in the vehicle-treated rats compared to the sham-operated rats (2.49 ± 0.15 vs. 1.21 ± 0.06 μg/g protein, *P* < 0.0001, Table 1). Compared with the omeprazole-treated rats, LV norepinephrine levels were significantly lower at the border regions in the rats treated with omeprazole and magnesium sulfate.

**Fig 4:**
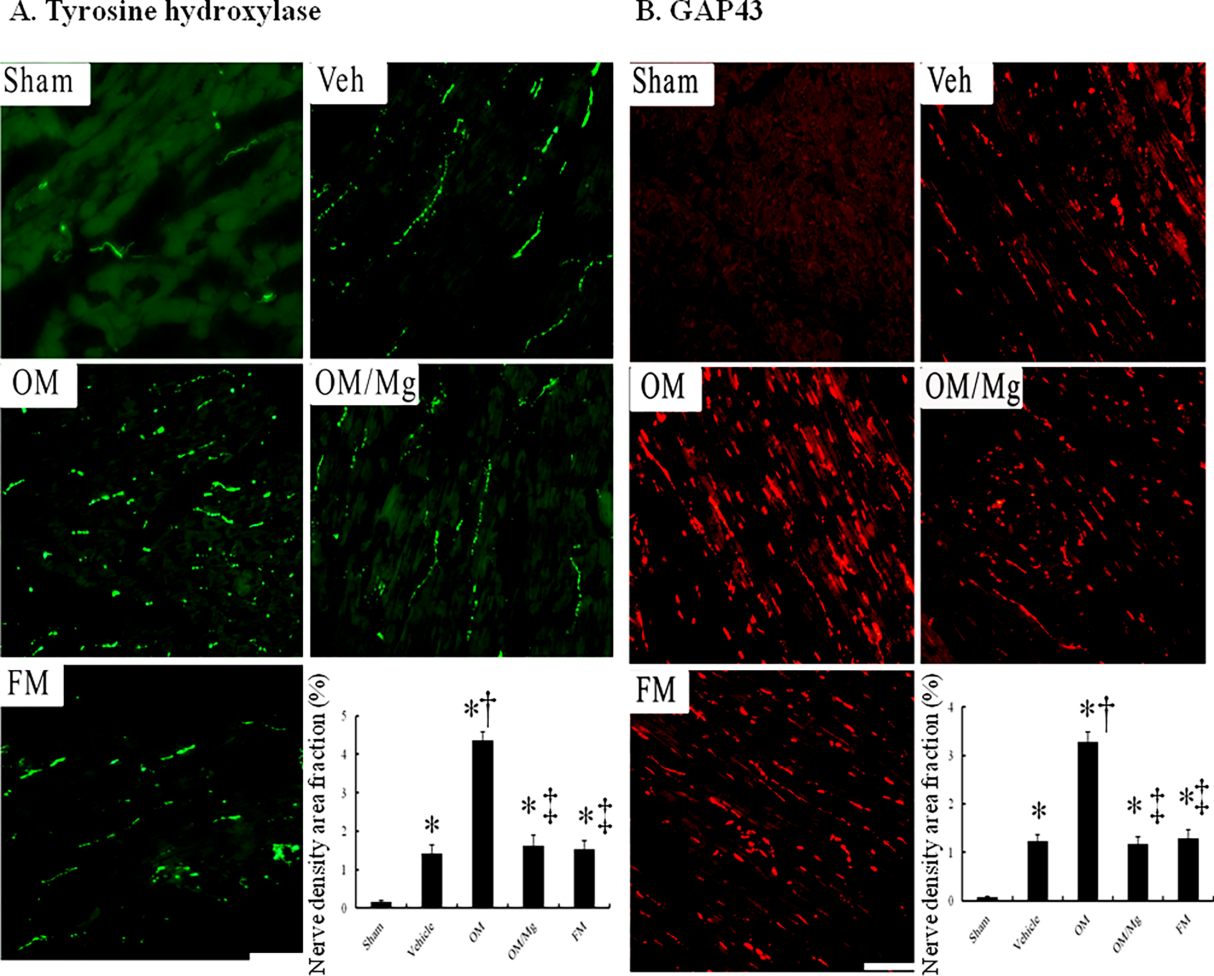
Immunofluorescent staining for tyrosine hydroxylase and GAP43 from the border regions (magnification 400×). (A) Tyrosine hydroxylase. Tyrosine hydroxylase-positive nerve fibers were located between myofibrils and were oriented in a longitudinal direction as that of the myofibrils. (B) GAP43. sham (n = 10); vehicle (n = 10); omeprazole (n = 9); omeprazole/MgSO_4_ (n = 11); famotidine (n = 10). Bar = 50 μm. Each column and bar represent mean ± SE. *P < 0.05, compared with the sham group; †*P* < 0.05, compared with the vehicle-treated rats; ‡*P* < 0.05, compared with the OM-treated rats.

The tyrosine hydroxylase-immunostained nerve fibers were oriented along the longitudinal axis of adjacent myofibers, and the tyrosine hydroxylase-positive nerve area fraction was significantly larger in the vehicle-treated infarcted rats than in the sham group (Fig 4A). The omeprazole-treated infarcted rats had larger nerve area fractions at the border regions compared with the vehicle-treated infarcted rats (4.35 ± 0.24% vs. 1.42 ± 0.23% in the vehicle group, *P* < 0.001), however this was reduced in the rats cotreated with magnesium sulfate (4.35 ± 0.24% vs. 1.62 ± 0.27% in the omeprazole + magnesium sulfate group, *P* < 0.001). Similar to the results of tyrosine hydroxylase, GAP43-positive (Fig 4B) nerve area fractions were significantly increased in the omeprazole-treated infarcted rats compared to those cotreated with magnesium sulfate.

### PPI treatment worsened ventricular arrhythmias

**Fig 5:**
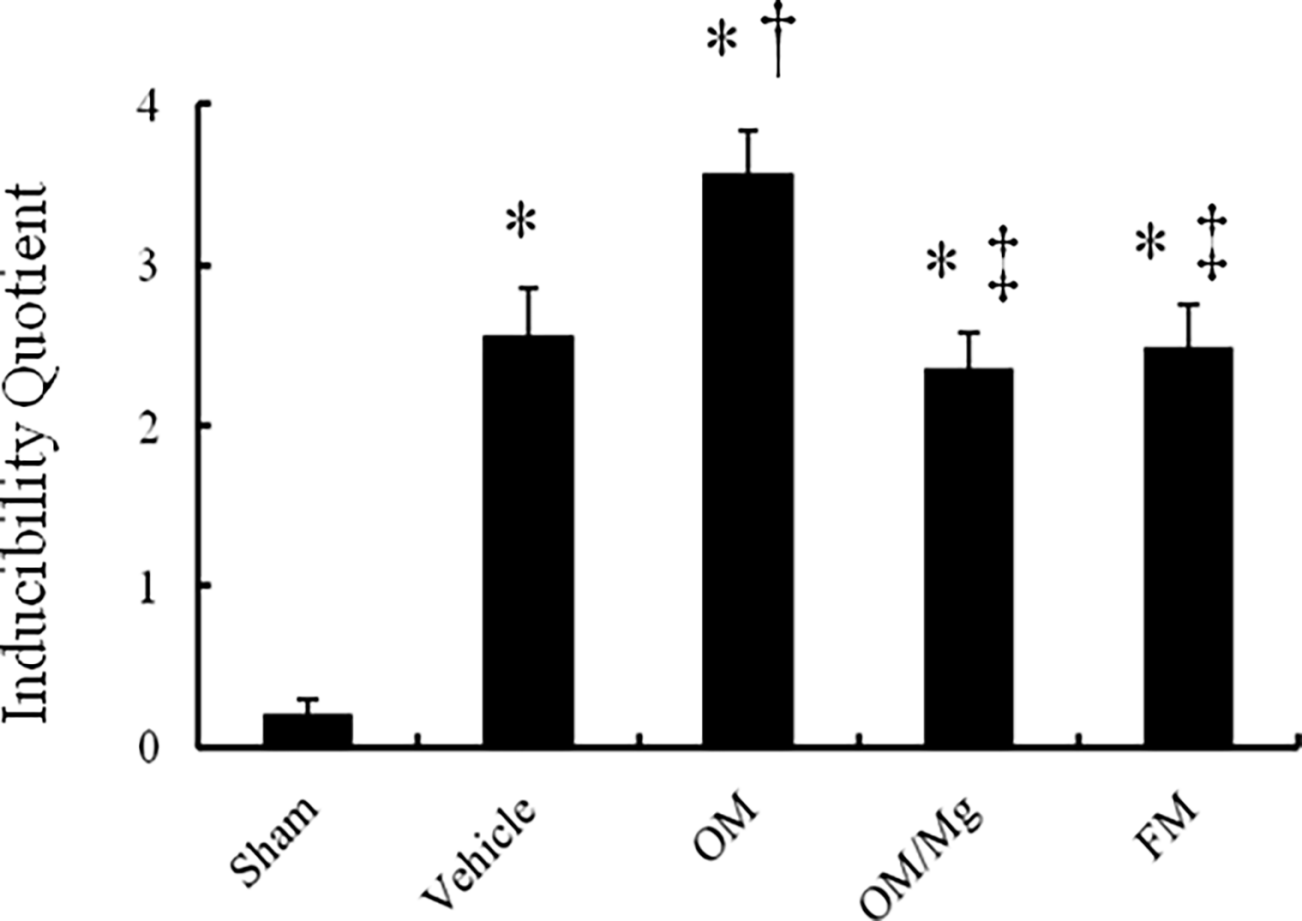
Inducibility quotient of ventricular arrhythmias by programmed electrical stimulation. *P < 0.05, compared with the sham group; †*P* < 0.05, compared with the vehicle-treated rats; ‡*P* < 0.05, compared with the OM-treated rats.

To assess the physiological impact of sympathetic reinnervation, programmed electrical stimulation was performed. The arrhythmia score in the sham-operated rats was very low (0.2 ± 0.1, Fig 5). In contrast, ventricular tachyarrhythmias, including ventricular tachycardia and ventricular fibrillation, were inducible by programmed stimulation in the vehicle-treated infarcted rats. The infarcted rats treated with omeprazole and magnesium sulfate had significantly decreased inducible ventricular tachyarrhythmias compared to those treated with omeprazole alone.

### PPI treatment increased NGF levels by enhanced superoxide signaling

**Fig 6:**
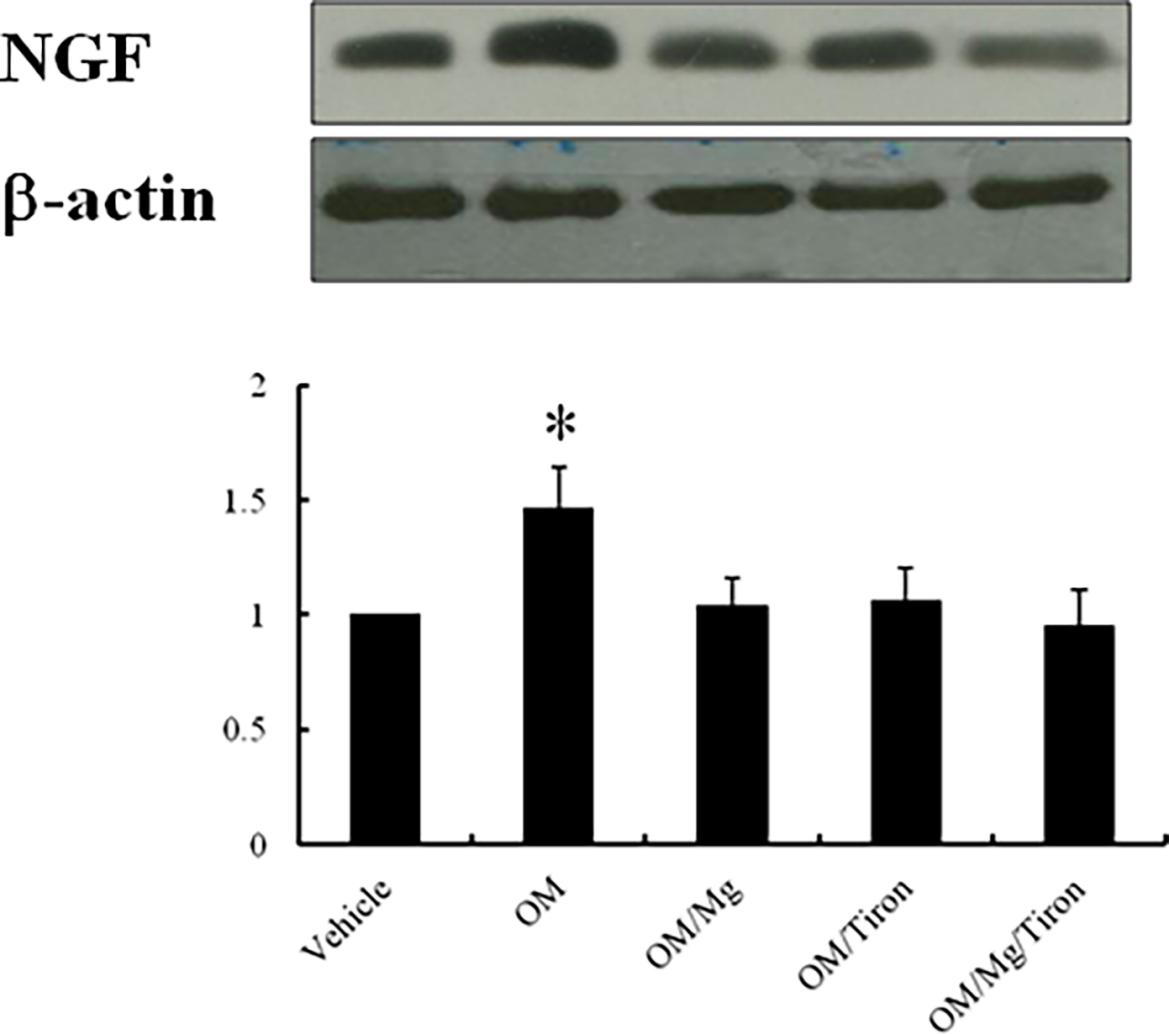
Experiment 2 (*ex vivo*). Western blot of NGF in the *ex vivo* study. In a rat isolated heart model, the increased NGF protein levels in response to omeprazole were inhibited by MgSO_4_ and the superoxide scavenger Tiron. Relative abundance was obtained by normalizing the density of NGF proteins to that of β-actin. Results are mean ± SE of three independent experiments. n = 5 in each group. *P < 0.05 compared with the vehicle-, omeprazole (OM) + MgSO_4_ (Mg)-, OM + Tiron-, and OM + Mg + Tiron-treated infarcted rats.

*Ex vivo* study. To further confirm the role of superoxide in modulating NGF levels, we used the superoxide scavenger Tiron. The coadministration of Tiron and the PPI abolished the effect of the PPI on NGF levels (Fig 6). Furthermore, the addition of lithium did not further attenuate NGF levels compared with the hearts treated with omeprazole + magnesium sulfate.

## Discussion

This is the first study to show that chronic treatment for 4 weeks with a PPI can lead to exaggerated sympathetic innervation through a magnesium-mediated ROS pathway after MI. The administration of magnesium could improve ventricular arrhythmias by scavenging ROS. These results are consistent with the detrimental effects of PPIs as documented structurally by impaired ventricular remodeling and increased cardiac nerve sprouting and DHE staining, molecularly by the myocardial NGF expression on mRNA and protein levels, biochemically by tissue superoxide, peroxynitrite and norepinephrine levels, pharmacologically by MgSO_4_ and Tiron, functionally by echocardiography, and electrophysiologically by exacerbation of fatal ventricular tachyarrhythmias. Our results are consistent with the findings of Weglicki et al [30], who showed a significant elevation in the cardiac tissue levels of neuropeptides associated with magnesium deficiency. Our data may provide a unifying mechanism for the association between the use of PPIs and worse ventricular arrhythmias after MI.

**Fig 7:**
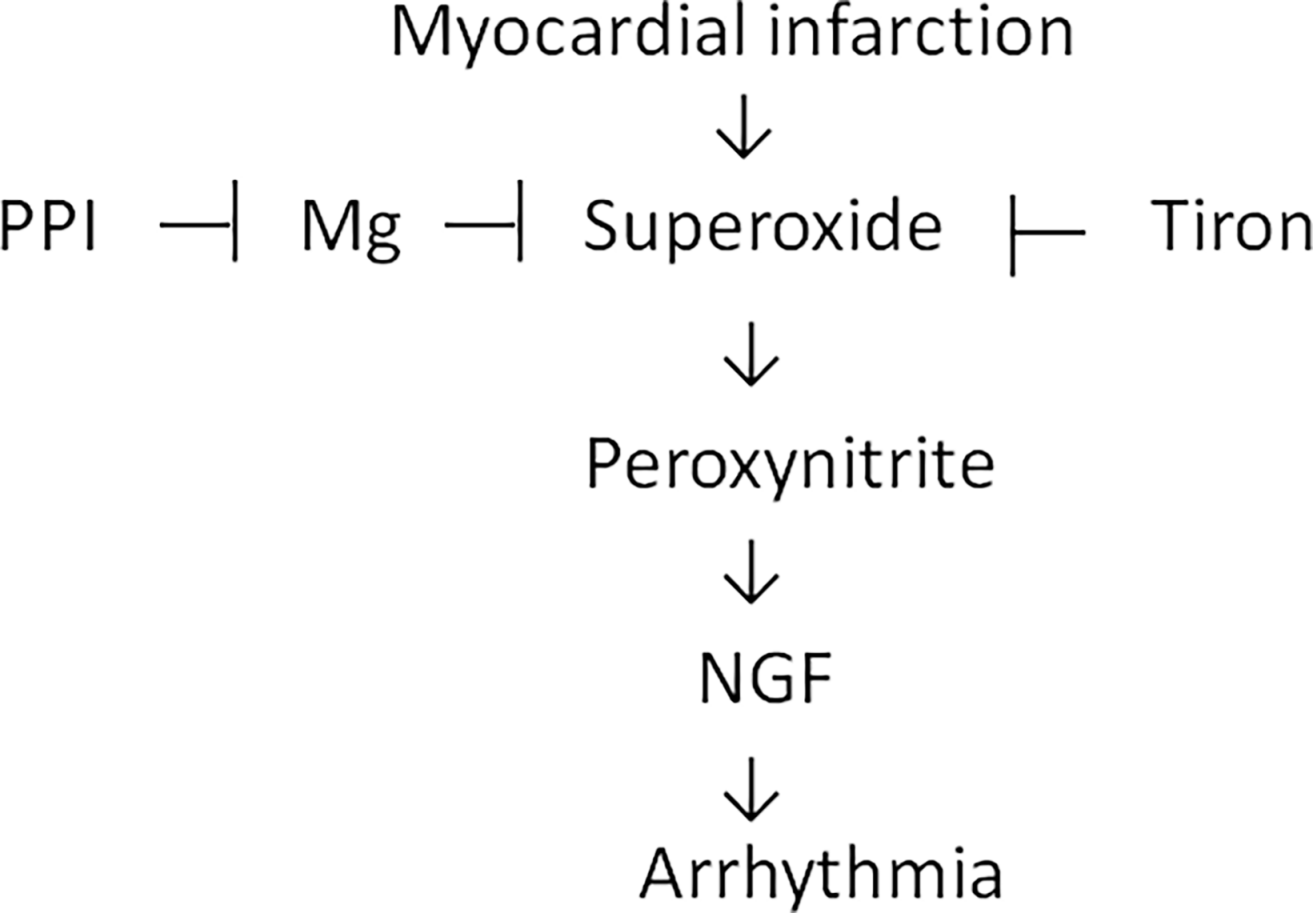
Reaction sequences leading to worsened arrhythmias by PPI administration. The diagram summarizes the immunofluorescent, molecular, and pharmacological evidence presented in this report. Inhibition of these signaling pathways by their respective inhibitors is indicated by the vertical lines.

The effect of PPI treatment on worsening ventricular arrhythmias was supported by three lines of evidence as shown in Fig 7.

1) The administration of low-dose omeprazole was associated with a decrease in myocardial Mg^2+^ during the 4 weeks of treatment, although serum magnesium levels did not significantly change compared with vehicle treatment. Measuring serum magnesium levels is the most common method to determine magnesium homeostasis; however, it does not accurately reflect total body magnesium storages because magnesium is predominantly localized to the intracellular compartment (99%). The intracellular concentration of Mg^2+^ has been shown to correlate well with the tissue concentration of Mg^2+^ but not with serum magnesium [31]. Thus, signs of magnesium deficiency can occur with normal serum levels [32]. This is consistent with the findings of Thongon et al [17], who reported no significant changes in serum Mg^2+^ concentration after 4 weeks of omeprazole treatment at 20 mg/kg per day in rats, a dose much higher than that used in this study. Given that no correlation has been reported between the concentration of serum magnesium and the concentration of tissue magnesium [33], it is not surprising that hypomagnesemia may be not present in patients with chronic depletion of Mg^2+^. These findings may explain our results of the beneficial effect of administering Mg^2+^ in the omeprazole-treated infarcted rats which appeared to be normomagnesemic.

Various mechanisms have been proposed to explain the low tissue levels of magnesium in rats administered with PPIs, including disturbance of tight junction function in the paracellular pathway, and transient receptor potential melastatin 6 channel function in the transcellular active pathway [34]. In addition, Ben-Ghedalia et al [35] reported that the elevation of luminal pH after the administration of PPIs led to a lower soluble concentration of Mg^2+^ from 79.61% at pH 4.4–5.15 to 8.71% at pH 7.8–8.15, and that this affected intestinal Mg^2+^ absorption.

2) Reduced myocardial Mg^2+^ content was associated with increased myocardial superoxide levels, which could be reversed by administering magnesium. Mg^2+^ has been reported to play a positive role in controlling ROS generation, and the administration of MgSO_4_ was shown to decrease superoxide dismutase in rats [36]. The direct molecular mechanisms for how Mg^2+^ acts as an antioxidant agent have yet to be fully elucidated. The O_2_^•−^ anions formed during post-MI ventricular remodeling may react with magnesium cations as O_2_^2-^ ions in electron transfer reactions leading to magnesium-oxygen products [37]. Furthermore, Mg^2+^ ions have been shown to destabilize neutrophil NADPH oxidase by ATP, and thereby prevent the generation of O_2_^•−^ radicals [38]. Thus, the depletion of magnesium cations may increase superoxide anions for lack of scavenging and preventing superoxide anions.

3) The administration of omeprazole was associated with worsened ventricular arrhythmias after MI. The influence of omeprazole on ventricular arrhythmias has not been investigated before. In this study, the PPI increased NGF levels through a superoxide-dependent pathway, and the ability of the superoxide scavenger Tiron to inhibit PPI-stimulated myocardial NGF levels strongly supports that superoxide anions had a mechanistic role. Furthermore, the addition of MgSO_4_ did not further attenuate NGF levels in the hearts treated with omeprazole + Tiron, implying that MgSO_4_ and Tiron share a common pathway to regulate NGF. Peroxynitrite, the reaction product of nitric oxide and superoxide, has been shown to activate activator protein-1 [16], which in turn can activate the *NGF* promoter and enhance the transcripts of *NGF*. These results extend our previous findings that antioxidation caused by the administration of N-acetylcysteine or xanthine oxidase inhibitors can attenuate sympathetic hyperinnervation after infarction [8,9].

### Other mechanisms

Although the present study suggests that the mechanisms of PPI-induced impairment of pacing-induced arrhythmias may be related to increased Mg^2+^-dependent superoxide production, other mechanisms of increasing free radicals after the administration of PPIs should also be considered. First, PPIs can interference the absorption of vitamin B_12_ [39] which is required for the conversion of homocysteine to cysteine, and elevated plasma homocysteine levels may increase ROS levels [40]. Second, a recent study reported that the administration of PPIs was associated with increased production of free radicals by impairing the lysosomal proton pump of the endothelium [41]. However, Tiron could reverse the increase in NGF levels, implying the role of superoxide in the PPI-mediated impact on NGF levels.

### Clinical implication

Safety issues associated with PPIs have recently attracted widespread attention. The present study provides strong evidence that PPIs have adverse effects on ventricular arrhythmias in an animal model. The oral dose of omeprazole used in this study (5 mg/kg/day in rats) is equal to an oral human dose of 40 mg on a body surface area basis [42]. Given that our studies were conducted using a clinical dose, the conclusions drawn in this study are clinically relevant. Whether or not the negative impact of PPIs on cardiovascular outcomes translates to clinically meaningful adverse events has been the subject of great debate. To date, no clinical research regarding impaired arrhythmias with the use of PPIs after MI has been reported.

In addition to a heterogeneous baseline in clinical patients, the duration of PPI treatment should also be considered. Landi et al [2] recently reported no significant difference in the risk of MI at 12 months between the use of a PPI and an H2 receptor blocker. The lack of a significant difference in cardiovascular risk in the short-term use is not surprising. Our results showed decreased myocardial Mg^2+^ content after 28 days (4 weeks) of omeprazole treatment in rats, which is about two human years, and this should be of concern, especially in patients who continuously use PPIs and diuretics. The US Food and Drug Administration released a warning about low magnesium levels associated with long-term PPI use in 2011 [43]. Given the potential complications of PPIs, we suggest that patients with a proven indication for a PPI should continue to receive it in the lowest effective dose, and that chronic PPI therapy should be discouraged.

### Study limitations

The limitations of the study have to be acknowledged. First of all, given its very short half-life at physiological pH, peroxynitrite precluded direct measurement in biological systems. Therefore, to estimate peroxynitrite formation, we assessed the most widely accepted marker of peroxynitrite, free nitrotyrosine, probably underestimating cardiac peroxynitrite formation [44]. Second, the precise source of increased ROS generation cannot be determined from this study. A number of cell types in the myocardium may be responsible for the increased levels of tissue ROS, such as endothelium and fibroblasts [45]. However, DHE staining was mainly observed in the cardiomyocytes compared with vascular smooth muscle and endothelial cells, suggesting that the cardiomyocytes were one of the main sources for cardiac ROS generation. Finally, the results with the use of animals as models of humans don't always translate to humans. Experimental MI models with permanent coronary occlusion fail to represent the clinical setting of an MI with late patency of the infarcted-related artery. Thus, its clinical results will require further studies to examine the effect of PPIs on the proarrhythmic action after MI.

## Conclusions

Our data show that infarcted hearts exposed to a PPI, but not an H2 receptor blocker, had decreased myocardial Mg^2+^ content, increased NGF expression, and consequently increased ventricular arrhythmias, probably through a superoxide-dependent pathway. Future studies are warranted to evaluate heart tissue Mg^2+^ levels to better monitor the side effects of the use of PPIs.
